# A Non‐Coding Oligonucleotide Recruits Cutaneous CD11b^+^ Cells that Inhibit Thelper Responses and Promote Tregs

**DOI:** 10.1002/advs.202400260

**Published:** 2024-06-19

**Authors:** Kahkashan Kamal, Elina Richardsdotter‐Andersson, Aleksandra Dondalska, Marie Wahren‐Herlenius, Anna‐Lena Spetz

**Affiliations:** ^1^ Department of Molecular Biosciences The Wenner‐Gren Institute Stockholm University Svante Arrhenius väg 20C Stockholm SE‐106 91 Sweden; ^2^ Department of Medicine Karolinska University Hospital Karolinska Institutet Visionsgatan 18, L8 Solna SE‐171 76 Sweden

**Keywords:** antigen‐presenting cells, cytokines, oligonucleotides, programmed‐death ligand 1, skin, T helper cells

## Abstract

Skin‐resident antigen‐presenting cells (APC) play an important role in maintaining peripheral tolerance via immune checkpoint proteins and induction of T regulatory cells (Tregs). However, there is a lack of knowledge on how to expand or recruit immunoregulatory cutaneous cells without causing inflammation. Here, it is shown that administration of a non‐coding single‐stranded oligonucleotide (ssON) leads to CCR2‐dependent accumulation of CD45^+^CD11b^+^Ly6C^+^ cells in the skin that express substantial levels of PD‐L1 and ILT3. Transcriptomic analyses of skin biopsies reveal the upregulation of key immunosuppressive genes after ssON administration. Functionally, the cutaneous CD11b^+^ cells inhibit Th_1/2/9_ responses and promote the induction of CD4^+^FoxP3^+^ T‐cells. In addition, ssON treatment of imiquimod‐induced inflammation results in significantly reduced Th_17_ responses. It is also shown that induction of IL‐10 production in the presence of cutaneous CD11b^+^ cells isolated after ssON administrations is partly PD‐L1 dependent. Altogether, an immunomodulatory ssON is identified that can be used therapeutically to recruit cutaneous CD11b^+^ cells with the capacity to dampen Th cells.

## Graphical abstract

In this article, Kamal et al. elucidate the immunoregulatory role of an oligonucleotide (ssON) that is found to recruit cutaneous immunoregulatory APCs with increased immune checkpoint protein expression. Functional T‐cell assays reveal that such immunoregulatory APCs inhibit Thelper cell responses and promote CD4^+^FoxP3^+^Tregs. This study envisions the use of ssON as therapeutic for rebalancing overactive Thelper cell responses in the skin.

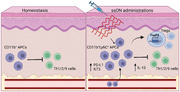

## 1 Introduction

Maintenance of the homeostatic cutaneous environment is dependent on a fine‐tuned cellular network acting in concert to prevent over‐reactive immune responses due to constant exposure to bacteria and antigens. Dysregulated T‐cell responses to external stimulus, injury, or pathogens have been implicated in the pathophysiology of chronic cutaneous inflammatory disorders such as atopic dermatitis (AD), and psoriasis.^[^ ^1^, ^2^, ^3^ ^]^ In addition to T‐cell activation, a multitude of cutaneous cells are involved in the development of skin lesions in AD. Such lesions involve complex interactions between different immune cells including tissue‐resident memory and recirculating T‐cells, different APC subsets, keratinocytes, and mast cells.^[^ ^2^, ^4^, ^5^ ^]^ The critical role of T‐cells in AD and several other inflammatory skin diseases is widely known. T‐cell co‐signaling pathways regulate T‐cell activation, where co‐signaling molecules are essential for determining the magnitude of the T‐cell response. It was suggested that excessive co‐stimulation and reduced inhibition of T‐cells by adjacent APCs can lead to a break in self‐tolerance, culminating in disease progression in, for example, AD.^[^ ^6^ ^]^ The programmed death‐ligand 1/programmed cell death protein 1 (PD‐L1/PD‐1) axis and other checkpoint receptors play a crucial role in the induction of peripheral tolerance via complementary mechanisms such as deletion or anergy of self‐reactive T‐cells.^[^ ^7^, ^8^, ^9^ ^]^ A recent study even suggested that PD‐1 is pivotal in maintaining peripheral tolerance toward cutaneous antigens and allowing antigen‐specific effector CD8^+^ T‐cells to co‐exist with antigen‐expressing cells without causing immunopathology.^[^ ^10^ ^]^ Immunoglobulin‐like transcript 3 (ILT3/LILRB4) belongs to a family of inhibitory receptors expressed on a variety of immune cells including dendritic cells, monocytes, macrophages, T‐cells, B‐cells, and natural killer cells.^[^ ^11^, ^12^, ^13^ ^]^ ILT3 can be induced by factors such as IL‐10, Vitamin D3, and type‐I interferons, providing a possible negative feedback loop to dampen immune responses after clearance of the danger signals in the microenvironment.^[^ ^11^ ^]^ The ILT3 receptor contains cytoplasmic immunoreceptor tyrosine‐based inhibitory motifs that can negatively regulate the activation of myeloid APCs rendering these APC tolerogenic with the capacity to induce differentiation of antigen‐specific regulatory CD4^+^ and CD8^+^ T‐cells.^[^ ^11^ ^]^

Although multifactorial and heterogenous causal mechanisms, dysregulated immune checkpoint signaling was reported in several inflammatory or autoimmune conditions.^[^ ^14^, ^15^, ^16^ ^]^ Previous reports have shown that mice deficient in the inhibitory molecule, PD‐L1, develop severe AD‐like symptoms after applications of the toll‐like receptor (TLR)7 agonist imiquimod.^[^ ^17^ ^]^ Moreover, PD‐L1 is highly expressed by a group of heterogeneous myeloid suppressor cells that can potently inhibit T‐cell responses and stimulate Treg responses.^[^ ^18^, ^19^ ^]^ Myeloid suppressor cells expand under pathological states such as cancer and inflammation^[^ ^20^ ^]^ and have been defined grossly by their expression of CD11b (integrin αM) and Gr1 in mice (anti‐Gr1 recognizes both Ly6C and Ly6G) as well as their functional capacity to suppress T‐cell responses.^[^ ^21^ ^]^ Notably, CD11b^+^Gr1^+^ cells with immune‐dampening functions exist in the spleen of healthy mice during steady‐state^[^ ^22^ ^]^ and can be expanded following TLR2‐ or TLR4‐mediated inflammation to limit cutaneous inflammation.^[^ ^23^ ^]^ However, there are still unanswered questions regarding the differentiation cues and functional properties of myeloid suppressor cells and their normal counterparts that maintain homeostasis.^[^ ^24^ ^]^

Previously, we reported that a 35‐nucleotide long ssON could temporarily inhibit TLR3/4/7 mediated endocytic pathway in monocyte‐derived dendritic cells and further demonstrated that ssON dampened dsRNA mediated inflammation in the skin of non‐human primates.^[^ ^25^ ^]^ Furthermore, we demonstrated that ssON prevented the degranulation of mast cells in response to certain ligands and alleviated skin‐related itch and inflammation.^[^ ^26^ ^]^ We also showed that the 35‐mer ssON binds to Nucleolin on the cell surface and provides anti‐viral activity against several viruses.^[^ ^27^, ^28^ ^]^ The 35‐mer ssON is non‐coding and does not target a specific sequence but requires a certain length of around 25–40 nucleotides to be fully active.^[^ ^28^ ^]^ However, there are still knowledge gaps regarding the immunological mechanisms underlying ssON's capacity to suppress inflammation in the complex tissue environment of the skin. In the present study, we aimed to gain further insights into ssON‐exposed APCs and their effects on T‐cell responses. We therefore injected ssON subcutaneously in mice, either as a single dose or daily for 4 days and characterized the cells at the injection site by transcriptomics and flow cytometry. We also explored their functional capacity and investigated whether they could inhibit T‐cell proliferation and/or alter cytokine production. Our findings unveil a new strategy in the form of an immunomodulatory non‐coding oligonucleotide that could be used to treat cutaneous autoimmune disorders to rebalance homeostasis in the skin by recruiting skin‐CD11b^+^Ly6C^+^ cells with upregulated PD‐L1 and ILT3 and capacity to inhibit Th_1/2/9/17_ responses and induce Tregs.

## 2 Results

### 2.1 Recruitment of Myeloid CD11b^+^Ly6C^+^ Cells to the Skin after Administration of ssON

**Figure 1:**
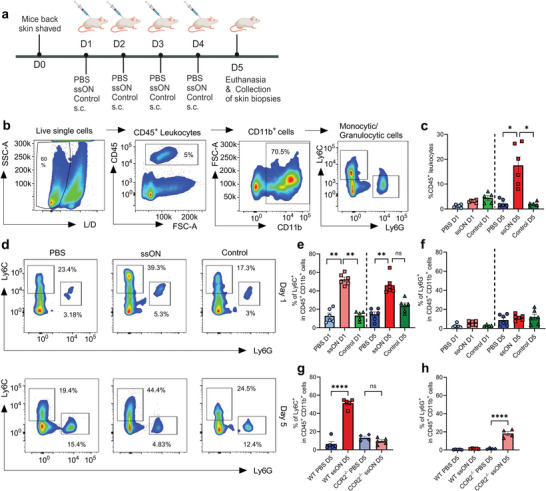
Accumulation of CD45^+^CD11b^+^ cells in the skin after injection with ssON. a) Experimental schedule. Created in BioRender. b) Single‐cell suspensions were prepared by enzymatic digestion and gentle dissociation of skin from the injection site. Gating strategy for the identification of myeloid cells. c) Frequency of CD45^+^ cells infiltrating the skin D1 and D5. d) Flow cytometry of CD11b^+^Ly6C^+^ and CD11b^+^Ly6G^+^ in the skin after single or multiple injections. e) Frequencies of CD11b^+^Ly6C^+^ and f) CD11b^+^Ly6G^+^ cells. g) Frequencies of CD11b^+^ Ly6C^+^ cells and h) CD11b^+^ Ly6G^+^ in WT and CCR2^−/−^ mice. Statistical significance differences between groups were measured by using the One‐way ANOVA test for non‐parametric and unpaired data (Kruskal Wallis test). Data are mean± SEM. *n* = 6 per group representative from >3 independent experiments. ^*^ *p* < 0.05, ^**^ *p* < 0.01, ^***^ *p* < 0.001.

To study immune cells in the skin after subcutaneous (s.c.) ssON administration, we performed flow cytometry analyses of cells isolated from murine skin biopsies collected from injection sites (**Figure** **1**a, gating strategy in 1b). Administration of ssON resulted in an increased frequency of cutaneous CD45^+^ leukocytes, which was significantly pronounced on day (D)5 but not on D1 (Figure 1c). We observed that the local injection site was occasionally accompanied by a visible lump but without any concomitant redness. The mild‐moderate cutaneous cellular infiltration was also observed in histology sections (Figure S1a,b, Supporting Information). We did not detect any increase in the frequency of CD3^+^ T‐cells or CD11b^+^CD11c^+^ dendritic cells after ssON injections (Figure S1c,d, Supporting Information). However, we found an increase in CD11b^+^SiglecF^−^Ly6G^−^CD11c^−^ cells that support a monocytic origin as murine skin conventional dendritic cells and Langerhans cells express CD11c, while eosinophils express SiglecF and granulocytes Ly6G.^[^ ^29^ ^]^ The CD11b^+^ cells were F4/80^+^ which further indicates a monocytic origin (Figure S1d, Supporting Information).

We found a significantly increased frequency of CD11b^+^Ly6C^+^Ly6G^−^ cells 24 h after the ssON injection (Figure 1d,e), providing additional support for a monocytic origin.^[^ ^29^, ^30^ ^]^ In contrast, there was no change in the frequency of CD11b^+^Ly6G^+^ granulocytic cells (Figure 1f). The increase of CD11b^+^Ly6C^+^ cells was also present after repeated ssON administrations on D5 (Figure 1e), while the frequency of CD11b^+^Ly6G^+^ cells remained constant (Figure 1f). The control oligonucleotide did not induce a significant increase in CD45^+^ leukocytes or CD11b^+^Ly6C^+^ cells. Thus, ssON administrations lead to the accumulation of CD11b^+^Ly6C^+^ monocytic cells locally in the skin upon both single and multiple exposures. Previous studies have shown that the recruitment of Ly6C^+^ monocytes is dependent on CCR2.^[^ ^30^ ^]^ To study whether the recruitment of cutaneous CD11b^+^Ly6C^+^ was dependent on CCR2, we injected CCR2^−/−^ mice with ssON. The frequencies of CD11b^+^Ly6C^+^ cells in the PBS groups were similar in the WT and CCR2‐deficient mice but the CCR2^−/−^ mice did not respond with an accumulation of CD11b^+^Ly6C^+^ cells after ssON injections (Figure 1g; Figure S2, Supporting Information). The CCR2^−/‐^ mice showed an increase in the frequency of CD11b^+^Ly6G^+^ cells after ssON injections (Figure 1h). Altogether, these data show that ssON administrations led to CCR2‐dependent recruitment of CD11b^+^Ly6C^+^ monocytic cells to the skin.

### 2.2 Upregulation of a Gene Expression Profile Associated with Immunoregulation after ssON Administrations

**Figure 2:**
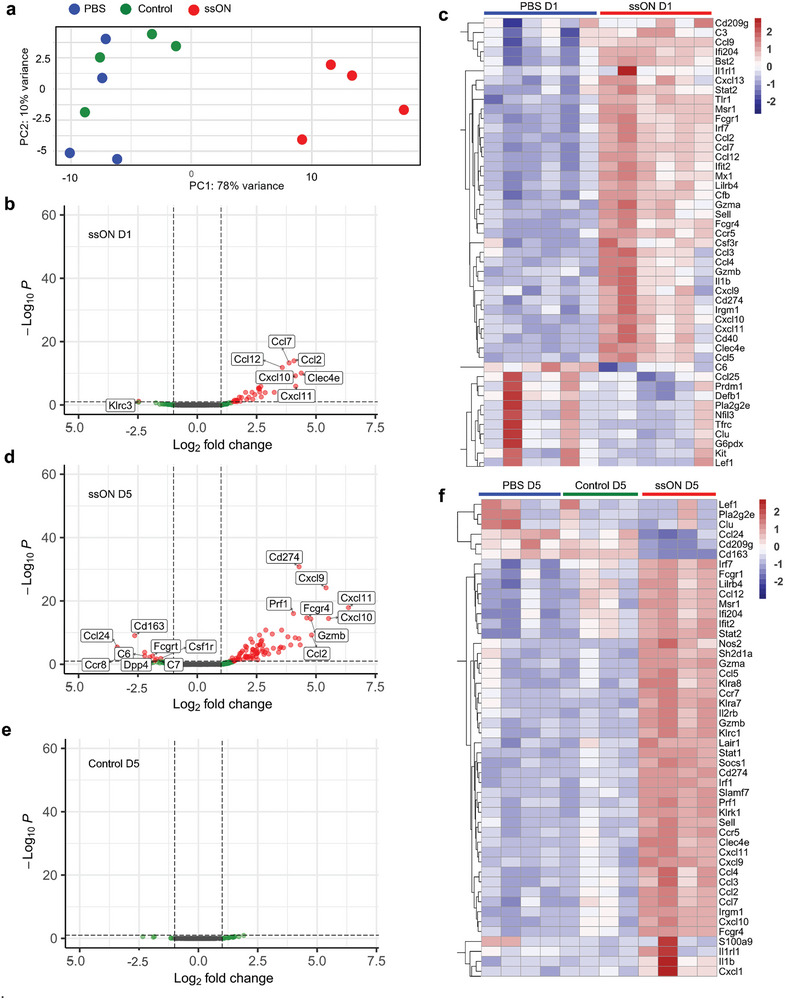
Transcriptomic analysis of ssON‐treated skin tissue reveals immunomodulatory changes. a) PCA of NanoString immune gene expression data from the skin of mice on D5 after repeated control oligonucleotide, ssON, or PBS injections. b) Volcano plots depicting the DEGs, and c) top 50 significantly up‐ or down‐regulated genes (adjusted *P*‐value < 0.05) in the skin on D1 after ssON‐treatment. d) Volcano plots showing the DEGs on D5 after ssON treatment and in (e), after injections with control oligonucleotide. The horizontal dotted line represents the threshold adjusted *P*‐value (<0.05) and the vertical dotted lines represent the threshold Log_2_fold change set at 1. f) Heatmaps of top 50 significantly up‐ or down‐regulated genes (*P*‐value < 0.05) in the skin of mice on D5 after ssON‐treatment or control oligonucleotide in comparison with PBS. *n* = 4–6 per group. Data from D5 is representative of 2 independent experiments.

To further gain insight into the mechanism of how ssON modulates the immune responses locally in the skin in vivo, we next assessed transcriptomic differences on D1 and D5. Principal component analyses (PCA) showed that the transcriptomic profiles of ssON‐treated skin samples clustered closely and differed from PBS skin samples on D5 (**Figure** **2**a). However, the control (15‐mer oligonucleotide) samples clustered closer to the PBS samples (Figure 2a,e).

Using the NanoString technology, we assessed the expression of 547 immune genes and found that 18 genes were significantly upregulated (Log_2_fold change>1 and adjusted *P*‐value < 0.05) on D1 (Figure 2b). In agreement with the CCR2‐dependent recruitment of CD11b^+^Ly6C^+^ cells, we detected upregulation of chemokines such as *Ccl2* that binds to CCR2.^[^ ^30^ ^]^ We also detected several other chemoattractants on D1 (Figure 2c), notably, ssON treatment resulted in significant upregulation of *Ccl2, Ccl3, Ccl5*, and *Cxcl10* which have been implicated in the recruitment of myeloid suppressor cells.^[^ ^18^, ^31^ ^]^ Altogether, transcriptomic data from biopsies collected on D1, indicate that ssON administration rapidly induces the production of chemokines which may recruit immunoregulatory cells.

On D5, the volcano plot (Figure 2d) shows 67 genes that were significantly differentially expressed in ssON‐treated skin samples in comparison to PBS‐treated (Log_2_fold change > 1 and adjusted *P*‐value < 0.05) of which 60 genes were upregulated and 7 were downregulated. A heatmap with the top 50 genes (DEGs) shows that *Pdl1 (Cd274)* was among the topmost genes significantly upregulated upon repeated administrations (Figure 2f). Among the other most significantly upregulated genes were *Irgm1, Cxcl10, Prf1*, and *Ifit2* with a Log_2_fold change > 4 (Figure 2d,f). Immunity‐related GTPase family M protein 1 (*Irgm1*) is a GTPase that regulates autophagy and mitochondrial homeostasis, furthermore, it is a master regulator of interferon signaling and protects against autoimmune disorders.^[^ ^32^, ^33^ ^]^ Amongst the downregulated genes were *Ccl24*, *Cd209g, Cd163, Ncam1(Cd56)*, and *Ccr8*. Downregulation of *Ccl24* (eotaxin for eosinophils)^[^ ^34^ ^]^ and *Ccr8* (implicated as a skin‐homing receptor for CD4^+^/CD8^+^ T‐cells and leukocytes in AD^[^ ^35^ ^]^) could theoretically be beneficial in the context of chronic inflammation. Additionally, the *Msr1* gene was significantly upregulated after both single and repeated administrations (Figure 2c,f). This gene encodes for CD204, which is a scavenger receptor‐1 class A expressed by M2‐like macrophages.^[^ ^36^ ^]^

To gain insight into how stromal cells respond to ssON treatment, we prepared single‐cell suspensions of skin biopsies and depleted CD45^+^ cells. NanoString RNA analyses showed that the CD45‐depleted cells responded with an upregulation of chemokines (Figure S3, Supporting Information).

**Figure 3:**
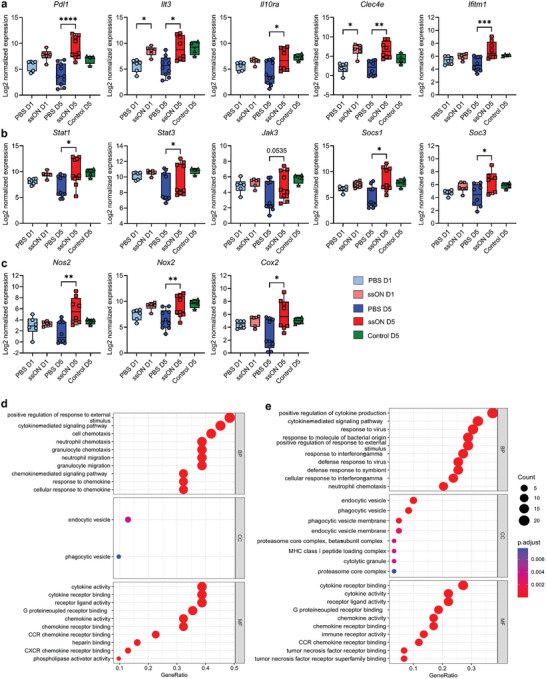
mRNA expression characteristics of cutaneous myeloid suppressor cell‐associated genes after single or repeated ssON injections. a) Log_2_ of normalized expression of genes *Pdl1*, *Ilt3*, *Il10ra*, *Clec4e*, and *Ifitm1*. b) *Stat1*, *Stat3*, *Jak3*, *Socs1*, and *Socs3*. c) *Nos2* (*iNos*), *Nox2*, and *Cox2*. d) GO term Analysis of differentially expressed genes showing top enriched pathways corresponding to biological process (BP), cellular component (CC), and molecular function (MF) after treatment with ssON on D1 and e) D5. Statistical non‐parametric analyses were made using the Mann–Whitney test, and multiple comparisons using One‐Way ANOVA (Kruskal–Wallis test). *n* = 4–10 mice per group. Data representative of 2 experiments on D5. ^*^ *p* < 0.05, ^**^ *p* < 0.01, ^***^ *p* < 0.001.

We next examined molecules specifically involved in modulating immune responses associated with myeloid suppressor cells and compared the Log_2_ normalized expression values on D1 and D5. *Pdl1* was significantly upregulated on D5 and there was a trend of upregulation on D1 (**Figure** **3**a). Similarly, *Ilt3* mRNA was significantly upregulated on both D1 and D5. There was also a significant upregulation of *Il10ra* expression, which is a crucial parameter associated with myeloid suppressor cell characterization.^[^ ^21^, ^37^ ^]^ *Clec4e*, which is another myeloid suppressor cell marker, was also found to be upregulated on both D1 and D5.^[^ ^38^ ^]^ Interferon‐induced transmembrane protein 1 (*Ifitm1*) is another top myeloid suppressor cell marker,^[^ ^38^ ^]^ and we indeed detected upregulation of *Ifitm1* on D5.

To get insights into signaling pathways, we investigated the mRNA levels of transcription factors and regulatory proteins such as *Stat1, Stat3, Jak3, Socs1*, and *Socs3* (Figure 3b). All these genes were found to be upregulated on D5, suggesting that JAK‐STAT pathways were activated. Notably, STAT1 and STAT3 are characteristic signaling pathways induced in myeloid suppressor cells,^[^ ^20^, ^24^ ^]^ and STAT3 is involved in the upregulation of PD‐L1.^[^ ^39^ ^]^ We also found that the suppressors of cytokine signaling 1 and 3 (*Socs1* and *Socs3*) were upregulated in the ssON‐treated skin biopsies. SOCS1 and SOCS3 are negative regulators of JAK/STAT pathways and suppress for example, IL‐6 production.^[^ ^40^, ^41^ ^]^ Hence, we detected upregulation of *Socs* in the ssON‐treated skin tissue providing a negative feedback loop in response to cytokine signaling. Interestingly, low‐level stimulation of JAK‐STAT signaling within tissue via cytokines and/or cell–cell interactions was recently reported to have a role in immune cell homeostasis.^[^ ^42^ ^]^

To gain insight into immunoregulatory factors, we studied the mRNA expression of enzymes such as inducible nitric oxide synthase (*Nos2*), NADPH oxidase 2 (*Nox2*), and prostaglandin‐endoperoxide synthase 2 (*Cox2*) which are well‐known to catalyze the production of reactive nitrogen species including ONOO^−^, reactive oxygen species (ROS) and prostaglandin E2, respectively.^[^ ^43^, ^44^, ^45^ ^]^ *Nos2* was found to be significantly upregulated in ssON‐treated skin on D5 (Figure 3c). NOS2 was shown to be involved in immunosuppression by direct nitration of T‐cell receptors thereby preventing activation and subsequent T‐cell proliferation.^[^ ^46^ ^]^ In addition, the expression of mRNA for ROS‐generating NOX2 was also upregulated moderately on D5 in ssON‐treated skin. Myeloid suppressor cell development was previously shown to be dependent on positive feedback between COX2 and prostaglandin synthesis.^[^ ^47^ ^]^ We indeed found that *Cox2* was significantly upregulated after ssON administrations (Figure 3c).

Furthermore, we performed gene ontology (GO) analysis of ssON‐induced signature genes, using the EnrichGo function (Figure 3d,e). On D1, some of the top GO terms were “positive regulation of response to external stimulus”, “cell chemotaxis, and ‘response to chemokines”. The cell component enriched GO term was “endocytic vesicle” which agrees with our earlier report showing that ssON interferes with endosomal pathways.^[^ ^25^ ^]^ Molecular function‐related GO terms that were enriched were “cytokine activity” and “cytokine receptor binding”. On D5, the top GO terms about biological processes were “positive regulation of cytokine production”, “response to interferon‐gamma”, and “response to virus”. Taken together, the transcriptomic data show that key immunoregulatory genes were upregulated after a single injection with ssON and that the effect was even more pronounced after multiple exposures.

### 2.3 Increased Expression of PD‐L1 and ILT3 on Myeloid Cells after ssON Administrations

**Figure 4:**
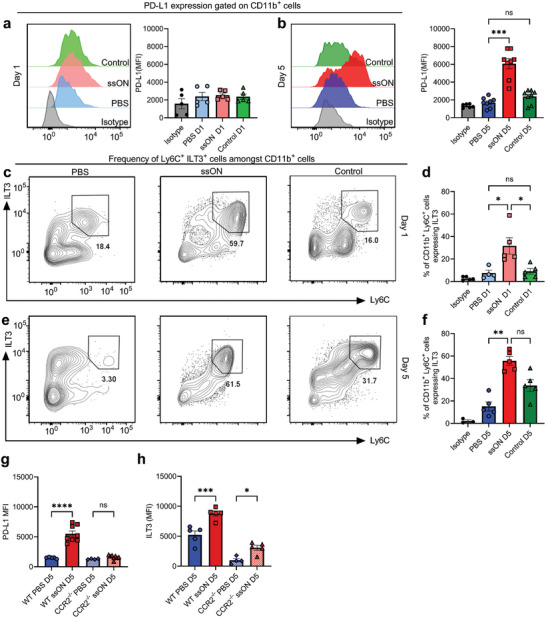
ssON injections result in the upregulation of PD‐L1 and ILT3 on cutaneous CD11b^+^ cells. a) Expression of PD‐L1 on CD11b^+^ skin cells on D1 and b) D5. c) Contour plots showing the frequency of Ly6C^+^ILT3^+^ cells in cutaneous CD11b^+^ cells after treatments with PBS, control oligonucleotide or ssON on D1 and e) D5. Frequencies of CD11b^+^Ly6C^+^ cells expressing ILT3 on d) D1 and f) D5. g) PD‐L1 and h) ILT3 expression in CD11b^+^ cells from WT and CCR2^−/−^ murine skin after repeated PBS or ssON injections. Data are mean ±SEM. *n* = 5 per group representative from >2 independent experiments. Pairwise comparisons were made by non‐parametric Mann‐Whitney test and multiple comparisons were made using non‐parametric One‐way ANOVA (Kruskal Wallis test with Dunn's multiple comparison test). ns non‐significant, ^*^ *p* < 0.05, ^**^ *p* < 0.01, ^***^ *p* < 0.001, ****p < 0.0001.

Next, we measured whether the CD11b^+^ skin cells expressed inhibitory PD‐L1 and ILT3 to validate the transcriptomic data. We could not detect PD‐L1 expression after a single ssON administration on D1 (**Figure** **4**a). However, flow cytometry revealed significantly increased levels of PD‐L1 on CD11b^+^ cells after repeated exposures of ssON on D5 (Figure 4b).

ILT3 is a key molecule that is expressed by myeloid suppressor cells and other tolerogenic cells.^[^ ^48^ ^]^ We found that ssON administration led to rapid upregulation of ILT3 on CD11b^+^ Ly6C^+^ cells (Figure 4c,d), which was even more pronounced on D5 (Figure 4e,f). Upregulation of PD‐L1 on CD11b^+^ cells was dependent on CCR2, indicating that recruited cells primarily expressed PD‐L1 on D5 (Figure 4g). Interestingly, the upregulation of ILT3 expression was only partially dependent on CCR2 (Figure 4h). Taken together, these results show a rapid upregulation of ILT3 on CD11b^+^Ly6C^+^ cells, while the kinetics of PD‐L1 expression was slower.

### 2.4 Cutaneous CD11b^+^ Cells Inhibit T‐Cell Proliferation In Vitro

**Figure 5:**
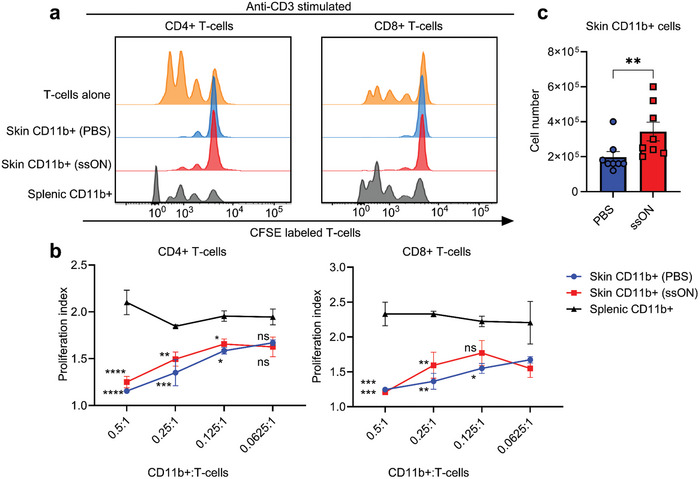
Cutaneous CD11b^+^ cells inhibit anti‐CD3‐mediated T‐cell proliferation. a) Histograms showing CFSE‐labeled T‐cells undergoing proliferation after anti‐CD3 stimulation in either the absence (orange) or the presence of CD11b^+^ cells from skin injected with PBS (blue) or ssON (red). The anti‐CD3 stimulated T‐cells were co‐cultured with splenic CD11b^+^ cells as a control (grey). b) Proliferation indices representing the frequencies of CD4^+^ and CD8^+^ T‐cells undergoing division in varying ratios of CD11b^+^ cells. Cutaneous cells were pooled *n* = 5 from each group. c) Number of CD11b^+^ cells obtained D5 from the skin of mice injected with PBS or ssON. The trypan blue method was used to count the cells and each data point represents cell numbers (mean ± SEM) obtained from *n* = 5 mice in each group. Data from one experiment is shown here out of three independent experiments. Multiple comparisons were made using a two‐way ANOVA test (Tukeys's test). ^****^ *p* < 0.0001, ^***^ *p* < 0.001, ^**^ *p* < 0.01, ^*^ *p* < 0.05, and ns *p* > 0.05.

To gain insight into the functional capacity of skin immunoregulatory cells on T‐cell responses, we next evaluated the effect of cutaneous CD11b^+^ cells on anti‐CD3‐mediated ligation of autologous CD4^+^ and CD8^+^ T‐cell proliferation. CFSE‐labeled splenic T‐cells were cultured on anti‐CD3 coated plates, in the absence or presence of cutaneous CD11b^+^ cells isolated from the PBS‐treated skin, ssON‐treated skin, or control CD11b^+^ splenocytes, for 72 h. In the absence of APCs, the immobilized anti‐CD3 antibody stimulated the proliferation of both CD4^+^ and CD8^+^ T‐cells, as shown in **Figure** **5**a. Notably, CD11b^+^ cells from both PBS‐ and ssON‐treated skin prevented T‐cell proliferation of both CD4^+^ and CD8^+^ T‐cells, in agreement with a functional role of steady state skin‐APC in maintaining homeostasis in the absence of pathogen or danger signal recognition. The proliferation index was lowest at the highest concentration of cutaneous CD11b^+^ cells (0.5:1). This inhibitory effect was dependent on the number of cells added to the T‐cells and decreased subsequently with lower numbers of cutaneous CD11b^+^ cells (Figure 5b). However, the splenic CD11b^+^ cells supported the proliferation of T‐cells as shown by the enhanced percentage of CFSE^low^ cells and consistently high proliferation indices in all cell ratios (Figure 5a,b). Importantly, the ssON‐treated skin yielded a significantly greater number of immunomodulatory CD11b^+^ cells as compared to the PBS‐treated skin (Figure 5c), although they displayed similar functional capacity to inhibit T‐cell proliferation in vitro wherein cell numbers were equalized.

In summary, repeated administration of ssON in vivo led to a substantial increase in the number of cutaneous CD11b^+^ cells that functionally suppressed anti‐CD3‐mediated T‐cell proliferation.

### 2.5 Alteration of T‐Cell Cytokine Profiles toward IL‐10 Production by Cutaneous CD11b^+^ Cells Recruited after ssON Injections

**Figure 6:**
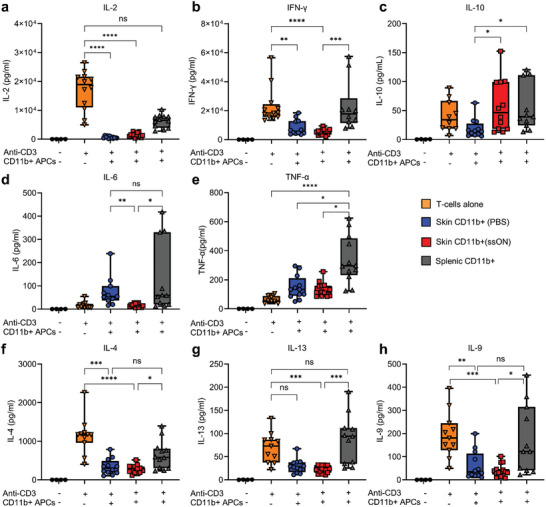
CD11b^+^ cells isolated from ssON‐treated skin promote IL‐10 production. Quantification of cytokines in cell culture supernatants obtained from co‐cultures with cutaneous CD11b^+^ cells and autologous T‐cells in the presence of anti‐CD3 stimulation. Cytokines levels of a) IL‐2, b) IFN‐γ, c) IL‐10, d) IL‐6, e) TNF‐α, f) IL‐4, g) IL‐13, and h) IL‐9. Data are mean ± SEM from 3 independent experiments, each well was run in duplicates. Comparisons between groups were made using the one‐way ANOVA test using the Kruskal–Wallis test. ^****^ *p* < 0.0001, ^***^ *p* < 0.001, ^**^ *p* < 0.01, ^*^ *p* < 0.05, and ns *p* > 0.05.

Next, we investigated if CD11b^+^ cells from ssON‐treated skin could drive T‐helper immune responses toward a certain Th subset. Here, we found that measurable levels of the pivotal T‐cell growth factor IL‐2 were significantly diminished in T‐cell co‐cultures with CD11b^+^ cells from either PBS‐ or ssON‐treated skin (**Figure** **6**a). The lack of IL‐2 is a likely explanation for the reduced T‐cell proliferation (Figure 5). This conclusion is corroborated by the finding that the addition of recombinant IL‐2 at the beginning of the cultures rescued CD4^+^ and CD8^+^ T‐cell proliferation in co‐cultures with cutaneous CD11b^+^ cells (Figure S4, Supporting Information).

We found that IFN‐γ levels were also reduced in the co‐cultures with cutaneous CD11b^+^ cells (Figure 6b). On the contrary, splenic CD11b^+^ cells did not inhibit the IFN‐γ production. Strikingly, CD11b^+^ cells from ssON‐treated skin induced a significantly higher production of the anti‐inflammatory cytokine IL‐10, compared to PBS‐treated skin (Figure 6c). Interestingly, an increase in IL‐10 was accompanied by a reduction of pro‐inflammatory IL‐6, in the supernatants from co‐cultures with ssON‐treated skin relative to PBS‐treated skin and splenocytes (Figure 6d). Splenic CD11b^+^ also contained cells that produced IL‐10 (Figure 6c), as expected.^[^ ^49^ ^]^ TNF‐α, another pro‐inflammatory cytokine produced by myeloid cells and activated Th_1_ cells, was abundantly produced in the cultures with CD11b^+^ cells from the spleen but at lower levels in co‐cultures with cutaneous CD11b^+^ cells (Figure 6e).

Figure 6f–h shows Th_2_ cytokines such as IL‐4 and IL‐13 as well as Th_9_ cytokine IL‐9 that were produced by anti‐CD3 activated T‐cells and their production was also equally high in co‐cultures with splenic CD11b^+^ cells. However, the T‐cell co‐cultures with CD11b^+^ cells from PBS‐ or ssON‐treated skin produced lower levels of these cytokines, with the co‐cultures containing CD11b^+^ cells isolated from ssON‐treated skin tending to have the lowest levels. We could not detect any significant production of these cytokines from CD11b^+^ cells cultured in the absence of T‐cells (Figure S5, Supporting Information).

Taken together, this data suggests that cutaneous CD11b^+^ cells exhibit an immunosuppressive function by depriving IL‐2, which is consistent with low T‐cell proliferation as shown in Figure 5, and expression of IL‐2 receptors on several subsets of skin‐APCs^[^ ^50^ ^]^ as well as increased expression of *Il2rb* after ssON administration (Figure 2f). Cutaneous CD11b^+^ cells isolated from either PBS‐treated or ssON‐treated cells significantly inhibited the production of Th_1/2/9_ cytokines but with the key difference that a higher number of these cells were isolated per mouse after ssON administrations. Additionally, ssON administration resulted in the infiltration of CD11b^+^ cells that supported IL‐10 production.

### 2.6 CD11b^+^ Cells from ssON‐Treated Skin Support Induction of FoxP3^+^ Tregs and PD‐L1‐Dependent IL‐10 Production

**Figure 7:**
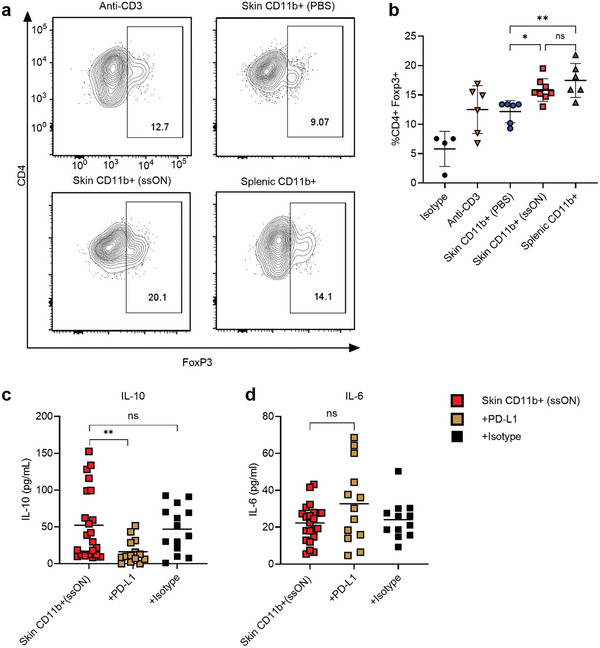
Induction of CD4^+^FoxP3^+^ T‐cells and PD‐L1‐dependent IL‐10 production by cutaneous CD11b^+^ cells after ssON treatment. a) Contour plots representing the intracellular staining of transcription factor FoxP3 in T‐cells derived from cultures as indicated in the presence of anti‐CD3 stimulation. b) Percentage of CD4^+^FoxP3^+^ T‐cells among T‐cells stimulated with anti‐CD3 alone, in the presence of CD11b^+^ cells isolated from the skin after injections with PBS or ssON, or CD11b^+^ cells from the spleen of BALB/c mice. Multiple comparisons were done using one‐way ANOVA, the Kruskal–Wallis test. c) Displays effects of blocking antibody PD‐L1 or isotype control in ssON‐derived skin CD11b^+^: T‐cell co‐cultures regarding IL‐10 production and d) IL‐6 production. An outlier was excluded in c using the outlier test (ROUTS method). Pairwise comparisons were made using the non‐parametric Mann–Whitney test. Data are mean + SEM from 3 independent experiments. ^****^ *p* < 0.0001, ^***^ *p* < 0.001, ^**^ *p* < 0.01, ^*^ *p* < 0.05, and *ns p* > 0.05.

IL‐10 is well known to suppress T‐cell responses but can also promote the development of Tregs.^[^ ^51^ ^]^ Although we observed IL‐10 production in co‐cultures with cutaneous CD11b^+^ cells obtained from ssON‐treated skin samples it was unclear whether Tregs were induced. Albeit a modest induction of *Foxp3* was detected in skin biopsies after ssON administration (Figure S6, Supporting Information). To elucidate whether Tregs were induced, we performed intracellular staining for FoxP3 in co‐cultures with anti‐CD3 stimulated T‐cells (**Figure** **7**a) and observed that cutaneous CD11b^+^ cells isolated after ssON administration induced a higher frequency of CD4^+^FoxP3^+^ T‐cells compared with PBS‐treated skin (Figure 7b). There was also a relatively high frequency of FoxP3^+^ cells in the co‐cultures with splenic CD11b^+^ cells in agreement with previous reports.^[^ ^49^ ^]^

The PD‐L1/PD‐1 axis has been implicated in the immunosuppressive function of immunoregulatory cells by converting Th cells to Tregs^[^ ^52^, ^53^ ^]^ in which IL‐10 may play a role.^[^ ^54^, ^55^ ^]^ To investigate whether PD‐L1‐mediated signaling plays any role in the induction of IL‐10 production and the immunoregulatory cells obtained after ssON administration, we co‐cultured cutaneous CD11b^+^ cells with anti‐CD3 activated T‐cells in the presence of PD‐L1 blocking antibody or isotype control. The results show that IL‐10 production was at least partially mediated by PD‐L1 as reduced quantities of IL‐10 were detected in the presence of anti‐PD‐L1 (Figure 7c). Interestingly, IL‐6 production was modestly increased, even though not statistically significant, in the presence of the anti‐PD‐L1 blocking antibody (Figure 7d). Moreover, anti‐PD‐L1 did not have any effect on IL‐2 or IFN‐γ production (Figure S7, Supporting Information). Altogether, these findings show that ssON administration results in the recruitment of cutaneous CD11b^+^ cells with enhanced capacity to induce Tregs. Furthermore, these data show that the induction of IL‐10 production in cultures with cutaneous CD11b^+^ cells is partly PD‐L1‐dependent.

### 2.7 ssON Administration Ameliorates Imiquimod (IMQ)‐Induced IL‐17 Production in Skin T‐Cells

**Figure 8:**
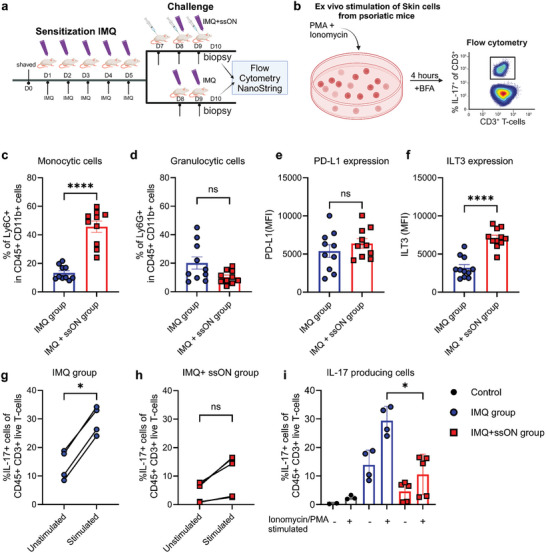
Inhibition of IMQ‐mediated Th_17_ responses in mice treated with ssON. a) Schematic representation of experimental scheme of IMQ‐induced psoriasis model in mice. Created in BioRender. b) Ex vivo stimulation of skin T‐cells with PMA and ionomycin. Frequency of c) CD11b^+^Ly6C^+^ monocytes and d) CD11b^+^ Ly6G^+^ granulocytes. Expression of e) PD‐L1 and f) ILT3 in cutaneous CD11b^+^ cells from IMQ‐treated mice. g) Ex vivo stimulation of skin cells with PMA/ionomycin from IMQ‐induced psoriatic mice and h) IMQ‐induced ssON‐treated mice. i) Comparison between groups. Data are representative of 2 independent experiments, pooled from several mice, *n* = 5–10 per group. Pairwise comparisons were made using the Mann–Whitney test. ****p < 0.0001, ^*^ *p* < 0.05, and *ns p* > 0.05.

To directly assess whether ssON affects Thelper responses in vivo, we applied IMQ‐containing cream to shaved murine skin daily for 5 days that induced inflammation with red and scaly skin (Figure S8a,b, Supporting Information). The mice were rested and then divided into two groups, one received ssON before challenging with IMQ and the other group received only IMQ challenges (**Figure** **8**a). We collected skin biopsies for NanoString RNA analyses and assessed IL‐17 production by flow cytometry after re‐stimulation with PMA and Ionomycin in comparison with medium control (Figure 8b). We observed an increased frequency of CD45^+^CD11b^+^Ly6C^+^ monocytic cells on D10 in the group treated with IMQ+ssON in comparison with the IMQ group, while similar frequencies of granulocytic cells were measured in both groups (Figure 8c,d). We did not detect any significant changes in PD‐L1 expression among groups (Figure 8e). However, we found a significant upregulation of ILT3 expression in the group that received ssON (Figure 8f). Flow cytometric analysis of T‐cells isolated from the IMQ group showed a high frequency of ex vivo production of IL‐17‐producing T‐cells, which was further enhanced by restimulation with PMA/Ionomycin (Figure 8g; Figure S8c, Supporting Information). There was a significant reduction in the frequency of IL‐17‐producing cells in the group that received ssON (Figure 8h,i; Figure S8d, Supporting Information). T‐cells isolated from untreated mice showed low production of IL‐17 as expected. Using NanoString, we evaluated the mRNA levels of inflammatory cytokines such as *Il1b, Il6*, and *Il17f* which were significantly reduced in the group that received ssON (Figure S8e, Supporting Information), confirming the efficacy of ssON in dampening the inflammation. There was also a trend of reduced *Il17*a and increased *Il10* expression. Altogether, these data show that ssON treatment can provide a dampening of Th_17_ responses in an inflammatory skin condition.

## 3 Discussion

The peripheral T‐cell repertoire in health encompasses T‐cell receptors with the potential to react toward self‐antigens, suggesting a key role for peripheral tolerance mechanisms to maintain homeostasis.^[^ ^7^, ^8^, ^9^ ^]^ Immune checkpoint proteins have been linked to the induction of peripheral T‐cell tolerance via clonal deletion and anergy even under physiological conditions in the skin.^[^ ^10^ ^]^ The upregulation of immune checkpoint proteins in our previous macaque study after injection of a non‐coding ssON led us to hypothesize that this ssON may be used to induce a local immune‐dampening environment (^[^ ^25^ ^]^ and Figure S9, Supporting Information). To elucidate this hypothesis, we herein injected ssON in the skin of mice and studied the local immune responses at the injection site. A high percentage of skin‐infiltrating CD45^+^CD11b^+^ cells detected after ssON injection(s), expressed Ly6C but lacked Ly6G, which is a phenotype associated with myeloid suppressor cells.^[^ ^21^ ^]^ Transcriptomic analyses of skin biopsies revealed an early upregulation of chemokines, and by using CCR2‐deficient mice, we demonstrated that recruitment of CD11b^+^Ly6C^+^ cells was CCR2‐dependent.

The cutaneous CD11b^+^ cells recruited after ssON injections expressed high levels of the inhibitory receptors ILT3 and PD‐L1. Notably, the kinetic analyses revealed a more rapid upregulation of ILT3 which was detected already on D1 while upregulation of PD‐L1 required repeated administrations. In addition, the upregulation of ILT3 on CD11b^+^ cells was only partially dependent on CCR2. The role of CCR2 in recruiting inflammatory monocytes is well established.^[^ ^56^ ^]^ However, reports have shown an involvement of CCR2 in the recruitment of myeloid suppressor cells to the skin during wound healing.^[^ ^57^, ^58^ ^]^ Here, we provide data showing CCR2‐dependent recruitment of myeloid cells to the skin that can functionally dampen Thelper cells. The upregulation of PD‐L1 expression was dependent on the length of the oligonucleotide that we injected as the 35‐nucleotide‐long ssON was more efficient in inducing PD‐L1 expression compared with the 15‐mer control. A functional difference comparing the 35‐mer with the control 15‐mer agrees with our previous studies showing a length requirement of the oligonucleotide to possess an immunomodulatory role.^[^ ^25^, ^28^ ^]^

Transcriptomics revealed that repeated exposures to ssON led to the upregulation of negative regulators of interferon signaling such as *Socs1* and *Socs3* involved in negative regulation of cytokine responses. Furthermore, *Nos2 (iNos)*, *Cox2*, and *Nox2* were significantly upregulated after repeated ssON treatment. Lastly, we show that ssON‐recruited CD11b^+^ cells could functionally suppress the proliferation and shift the cytokine profile in anti‐CD3 activated T‐cell cultures as well as increasing FoxP3 expression. Altogether, these findings suggest that ssON administration results in the recruitment of CD11b^+^ cells in the skin with enhanced capacity to induce Tregs. Furthermore, our data show that the induction of IL‐10 production in T‐cell co‐cultures with cutaneous CD11b^+^ cells is PD‐L1 dependent, which is consistent with previous findings.^[^ ^54^, ^55^ ^]^

We showed that cutaneous myeloid CD11b^+^ cells assessed for functional antigen‐presenting capacity directly ex vivo skewed the T‐cell response to a more anergic state as shown by inhibition of CD8^+^ and CD4^+^ T‐cell proliferation. In contrast, CD11b^+^ splenic cells supported T‐cell proliferation as expected. The myeloid CD11b^+^ cells present in the skin of mice injected with PBS also showed potent inhibition of CD4^+^ and CD8^+^ T‐cells. Notably, we detected a significant increase in the number of CD11b^+^ cells isolated from ssON‐treated skin as compared with PBS‐injected skin, without concomitant redness of the skin. This makes us hypothesize that the CD11b^+^ cells that infiltrate in ssON‐treated skin might constitute an enrichment of cells that have a normal counterpart. Finally, we provided evidence that ssON treatments ameliorate Th_17_ responses in a cutaneous IMQ model.

In conclusion, our data support the hypothesis that ssON could be used as an immunomodulator to dampen local inflammation in the skin by the recruitment of myeloid suppressor cells with increased PD‐L1 and ILT3 expression and the capacity to promote Tregs.

## 4 Experimental Section

### Oligonucleotides

A 35 bases long fully phosphorothioate‐modified single‐stranded oligonucleotide (ssON35mer), with the sequence: 5′GAAGTTTTGAGGTTTTGAAGTTGTTGGTGGTGGTG‐3′ and the 15‐mer: 5′‐GGTTTTGAAGTTGTT‐3′, was purchased from Integrated DNA technologies.

### Mouse Experiments

Female BALB/cAnNCrl and C57BL/6J mice at 8–15 weeks (weighing 16–22 g) were obtained from Charles River Laboratories. Female B6.129S4‐Ccr2tm1Ifc/J (CCR2^−/−^, Strain #:004999) were purchased from the Jackson Laboratory. Mice had access to food and water ad libitum and were maintained in a 12 h light/dark cycle and always in groups. Cages were enriched with wood chips, a cardboard house or a roll, a wooden stick, paper, and a piece of cotton. All animal experiments were approved by the local ethical committee in Stockholm, Sweden. Animal experiments were performed in compliance with the ARRIVE guidelines for reporting animal research. On day 0, the back skin of the anesthetized animals was shaved. Oligonucleotides or PBS (200 µL) were subcutaneously injected on the back skin at 1 mg Kg^−1^ body weight while the animals were anesthetized with isoflurane. Eye gel was applied, and a heating pad was used while the animals were under anesthesia. Injections were carried out consecutively on days 1, 2, 3, and 4. On day 1 or day 5, animals were euthanized and skin biopsies were collected for downstream analysis.

### Flow Cytometry Analysis

Single‐cell suspensions were prepared from murine skin biopsies. The skin tissue was incubated overnight in Dispase II (Roche, 1 mg mL^−1^) and/or 1 h at 37 °C, 5% CO_2_ the following day. The biopsies were minced into small pieces and subjected to enzymatic dissociation for 30 min at 37 ˚C, 90 rpm. Enzyme mix composed of Liberase TM (0.125 U/mL) and DNAse (0.02 mg mL^−1^) (both from Roche) in RPMI medium containing HEPES 10 mm, 3% FCS, and 1% Penicillin/Streptomycin. Finally, the tissue was homogenized in C‐tube (Mylteni) using a gentleMACS dissociator and strained through a 70 µm cell strainer to obtain a single‐cell suspension. The cells were stained with Live Dead dye Aqua and antibodies for 30 min at 4 °C and washed with wash buffer. The samples were acquired on a BD FACSverse machine and all analysis was performed with FlowJo software (Tree Star). Antibodies/dyes that were used for cell surface staining are tabulated in Table S1 (Supporting Information) available in the Supporting Information.

### RNA Extraction

RNA was extracted from the tissue using the RNeasy Plus Mini kit (Qiagen) using the manufacturer's protocol. Briefly, the skin tissue was homogenized in RLT buffer with β‐mercaptoethanol (10 µL/mL), using Miltenyi's M tubes in Miltenyi's GentleMACS dissociator. Then the lysate was applied to gDNAse eliminator spin columns to eliminate the DNA and then washed extensively to elute the RNA. The RNA quality and concentration were measured using NanoDrop 8000 Spectrophotometer.

### RNA Extraction from CD45‐Depleted Skin Cells

Skin biopsies were subjected to enzymatic digestion to obtain single‐cell suspension. Cells were pooled from five mice and further subjected to immunomagnetic positive selection for CD45^+^ leukocytes using EasySep Mouse CD45 Positive selection kit (Catalog # 18945). The remaining fraction containing CD45‐depleted skin cells was spun down and the cell pellets were lysed with RLT buffer. The RNA from the cells was isolated using RNeasy Mini kit (Qiagen) using the manufacturer's protocol.

### NanoString Analysis

The nCounter Mouse Immunology Panel (Nanostring Technologies, Seattle, WA) was used to analyze gene expression in the RNA samples, wherein 100 ng was mixed with the reagents and hybridized at 65 °C overnight. The samples were run on the nCounter Prep Station. Quality control and data normalization (to housekeeping genes) were performed on the count data using the nSolver Analysis software 4.0 (NanoString Technologies). A detection threshold was set using the mean plus 3 standard deviations of the negative controls. Differential gene expression analysis was done using the DESeq2 package in R.^[^ ^59^ ^]^ PCA plots and volcano plots were constructed using R. Heatmaps were generated by regularized log transformation of the data and using the TopVarGenes function to visualize the most variable genes. Gene Ontology analysis was done to visualize enriched terms using the EnrichGo function.

### T‐Cell Proliferation Assay

The skin biopsies were enzymatically digested, and cells were pooled from 5–10 BALB/c mice from each group to get the required cell number. CD11b^+^ cells were isolated from skin cells by using the EasySep Mouse CD11b Positive Selection Kit II (Stem Cell Technologies). T‐cells were isolated from the spleen by using the EasySep Mouse T‐cell Negative Isolation Kit (Stem Cell Technologies). The T‐cells were labeled with CellTrace CFSE Cell Proliferation dye (Invitrogen) by incubating the cells with the dye (2.5 µm) for 8–10 min at room temperature. 96‐well round bottom plates were coated with anti‐CD3 antibody (5 µg mL^−1^, Clone 1452C11) for 2 h at 37 °C. The plates were washed once with 1X PBS to remove unbound antibodies. The CD11b^+^ cells were added to the wells at a ratio of 0.5:1, 0.25:1, 0.125:1, and 0.0625:1. Autologous CFSE labeled T‐cells from BALB/c mouse spleen were added to the wells (80 000/well). The plates were incubated for 3 days at 37 °C, 5% CO_2_. On day 4, cells were collected and stained with Live Dead marker (IR), anti‐CD4 and anti‐CD8. On certain occasions, anti‐PD‐L1 blocking antibodies (10 µg mL^−1^), isotype control (10 µg mL^−1^), or recombinant IL‐2 (Proleukin) (30 U/mL) were added to the wells. The data was analyzed using FlowJo software and the proliferation index was obtained by using the Proliferation modeling tool in the software.

### Cytokine Bead Array

The cell culture supernatants were collected from the resulting co‐cultures with T‐cells. The cytokines were quantified using a Mouse T Helper Cytokine Panel Version 3 (Cat#741044, LEGENDplex) kit from BioLegend using the manufacturer's instructions.

### FoxP3 Intracellular Staining

Invitrogen FoxP3/Transcription Factor Staining kit (Cat#00‐5523‐00) was used to permeabilize the cells. Briefly, the cells were first stained for the cell surface markers and a Live/Dead marker. After the final wash, the cells were incubated with fixation and permeabilization solution and incubated for 30–60 min at 2–8 °C. Cells were washed once in 1X permeabilization buffer and then stained with anti‐FoxP3 or isotype control antibody (1:400 dilution) added directly to the cells. After 30 min of incubation at room temperature, cells were washed again with 1X permeabilization buffer and resuspended in FACS wash buffer. Data was acquired on BD FACSverse and analyzed using FlowJo software.

### Imiquimod‐Induced Inflammation Model

Female BALB/cAnNCrl (8–10‐week‐old) mice were shaved on their backs and subjected to sensitization with 40 µL of 5% IMQ cream for five consecutive days. Thereafter, the mice were allowed to rest and on day 7, they were divided into two groups, where one group received ssON (1 mg Kg^−1^) injections s.c. on their backs, consecutively for 3 days (days 7–9). The mice in both groups were re‐challenged with 5% IMQ on days 8 and 9. On day 10, mice were photographed and euthanized. The skin biopsies were collected for downstream analysis by flow cytometry.

For ex vivo stimulation of skin T‐cells, skin biopsies were digested, and cells were pooled from five mice and stimulated with PMA (50 ng mL^−1^) and Ionomycin (750 ng mL^−1^) in the presence of BD GolgiPlug Protein Transport Inhibitor (containing Brefeldin A #555029) for 4 h. The cells were subjected to intracellular staining using the Intracellular Fixation & Permeabilization Buffer Set from ThermoFisher following manufacturers’ instructions.

### Ethics Statement

The present studies in mice were reviewed and approved by the Stockholm Ethical Committee on animal experiments, permit number 11436–2020, and followed the Directive 2010/63/European Union of the European Parliament and of the Council, The Swedish Animal Welfare Act [SFS (Svensk författningssamlingar) 1988:534], The Swedish Animal Welfare Ordinance (SFS 1988:539), and the regulations regarding the use of animals for scientific purposes: SJVFS (Statens jordbruksverks författningssamlingar) 2017:40 (L150) specifically according to 7 kap. §9 djurskyddslagen (2018:1192) and SJVFS 2019:10. Animal studies were performed with the implementation of the principles of the 3Rs. All personnel involved in the animal experiments were trained and accredited according to FELASA. The work was carried out to minimize discomfort, distress, and pain in the mice.

### Statistical Analysis

All data were analyzed using the GraphPad Prism software version 10. Pairwise comparisons between groups were made using the Non‐parametric Mann–Whitney test. Comparisons between multiple groups were made using the Non‐parametric One‐Way ANOVA Kruskal–Wallis test.

## Conflict of Interest

A.L.S. and A.D. declare ownership in TIRmed Pharma having IPR related to ssON. K.K., E.A.R., and M.W.H. declares no conflict of interest.

## Author Contributions

A.L.S. and K.K. conceptualized the study. K.K. performed all the in vivo and ex vivo experiments with skin APCs and T‐cells. A.D. standardized the mouse skin digestion protocols. E.A.R. performed the NanoString assay. K.K. performed the bioinformatic analyses. Data analysis was conducted by K.K. and A.L.S.. A.L.S. and M.W.H. supervised the study. K.K. and A.L.S. wrote the paper. All authors discussed the results and provided comments and feedback.

## Supporting information

Supporting Information

## Data Availability

Data sharing is not applicable to this article as no new data were created or analyzed in this study.
