# Supplementary material for: A Non‐Coding Oligonucleotide Recruits Cutaneous CD11b+ Cells that Inhibit Thelper Responses and Promote Tregs
Source: Adv Sci (Weinh). 2024 Jun 19;11(31):2400260. doi: 10.1002/advs.202400260 (PMC11336929; doi:10.1002/advs.202400260)
Supplement: Supplementary file 1 — Supporting Information [file ADVS-11-2400260-s001.docx]

Supporting Information

**A Non-Coding Oligonucleotide Recruits Cutaneous CD11b^+^ Cells that Inhibit Thelper Responses and Promote Tregs**

*Kahkashan Kamal*, Elina Richardsdotter-Andersson, Aleksandra Dondalska,* *Marie Wahren-Herlenius,* *Anna-Lena Spetz*^.^*

**Table S1. Antibodies/dyes that were used for FACS or functional analysis.**

| Reagent or Resource | Source | Clone/Catalog Number |
| --- | --- | --- |
| Anti-mouse CD45; dil 1:500 | BD Biosciences | Clone C30-F11 #564590 |
| Anti-mouse CD11b; dil 1:200 | BD Biosciences | Clone M1/70 #561098 |
| Anti-mouse Ly6C; dil 1:200 | BD Biosciences | Clone AL-21 #560595 |
| Anti-mouse Ly6G; dil 1:200 | BD Biosciences | Clone 1A8 #560602 |
| Anti-mouse PD-L1; dil 1:500 | BD Biosciences | Clone MIH5 # 564716 |
| Anti-mouse ILT3; dil 1:250 | Biolegend | Clone H1.1 # 144903 |
| Anti-mouse CD4; dil 1:100 | BD Biosciences | Clone RM4-5 #561091 |
| Anti-mouse CD8; dil 1:100 | BD Biosciences | Clone 53-6.7#563898 |
| Rat IgG2a, λ Isotype; dil 1:500 | BD Biosciences | Clone B39-4 #562965 |
| Armenian Hamster IgG Isotype | Biolegend | Clone HTK888 #400907 |
| LIVE/DEAD™ Fixable Aqua Dead Cell Stain | ThermoFisher | #L34957 |
| LIVE/DEAD™ Fixable Near-IR Dead Cell Stain | ThermoFisher | #L10119 |
| Ultra-LEAF™ Purified anti-mouse CD274 | Biolegend | Clone 10F.9G2 #124340 |
| Anti-mouse IL-17A; dil 1:200 | Biolegend | Clone TC11-18H10.1 #506921 |

Figure S1.


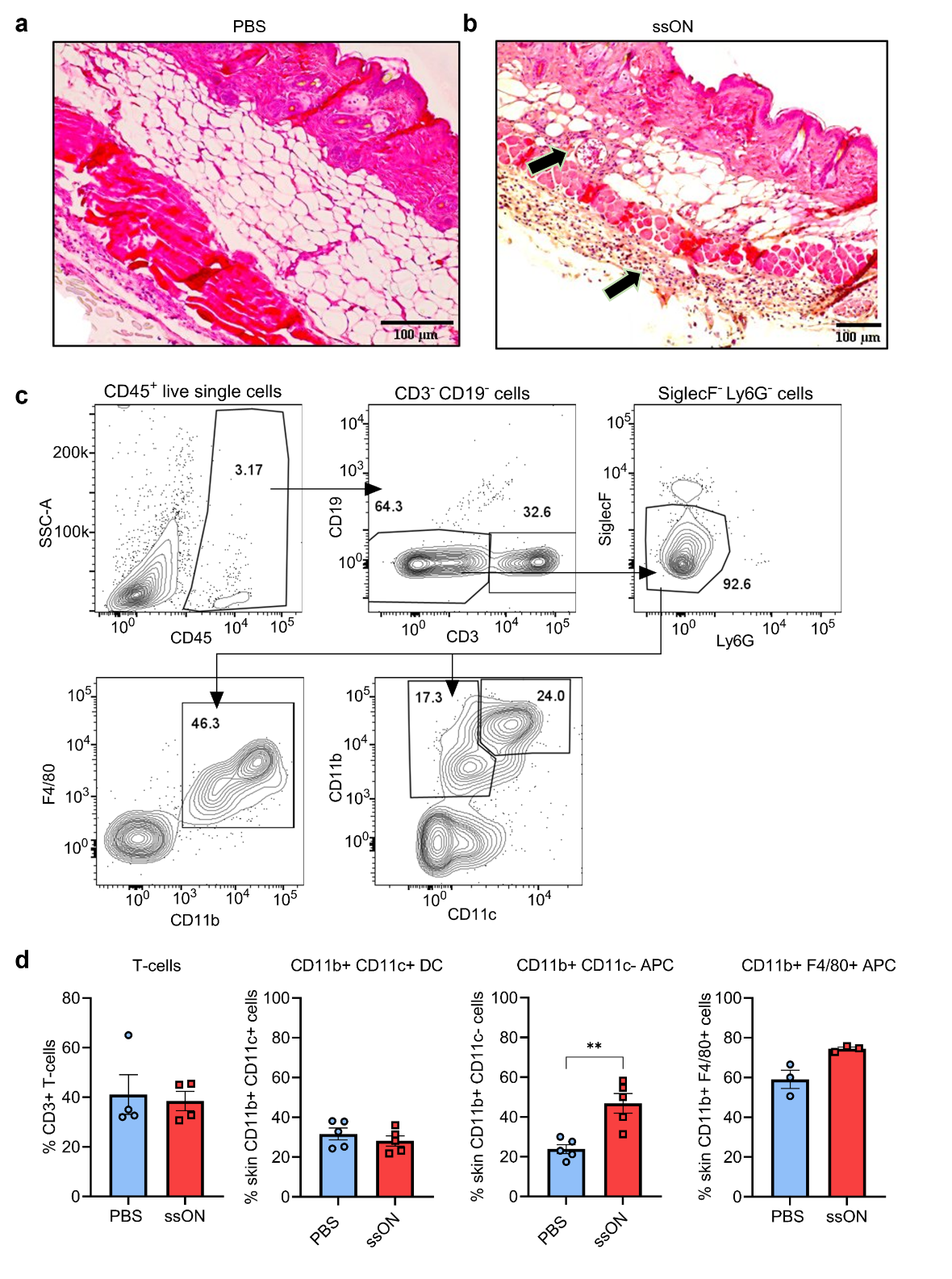


**Local infiltration of cells in the skin after ssON injections.** Hematoxylin-eosin staining of skin sections obtained from mice on D5 **a**) PBS injected group **b**) ssON injected group. Arrows pointing out local areas of cells. **c)** Gating strategy to identify the CD11b^+^ skin APC subsets. **d)** The frequencies of T-cells and CD11b^+^ subsets after ssON administration in the skin D1. Representative data was obtained by pooling two independent experiments with *n*=3-5 mice per group. Data are mean+SEM. Pairwise comparisons were made using the non-parametric Mann-Whitney test. ** *p*<0.01.

**Figure S2**


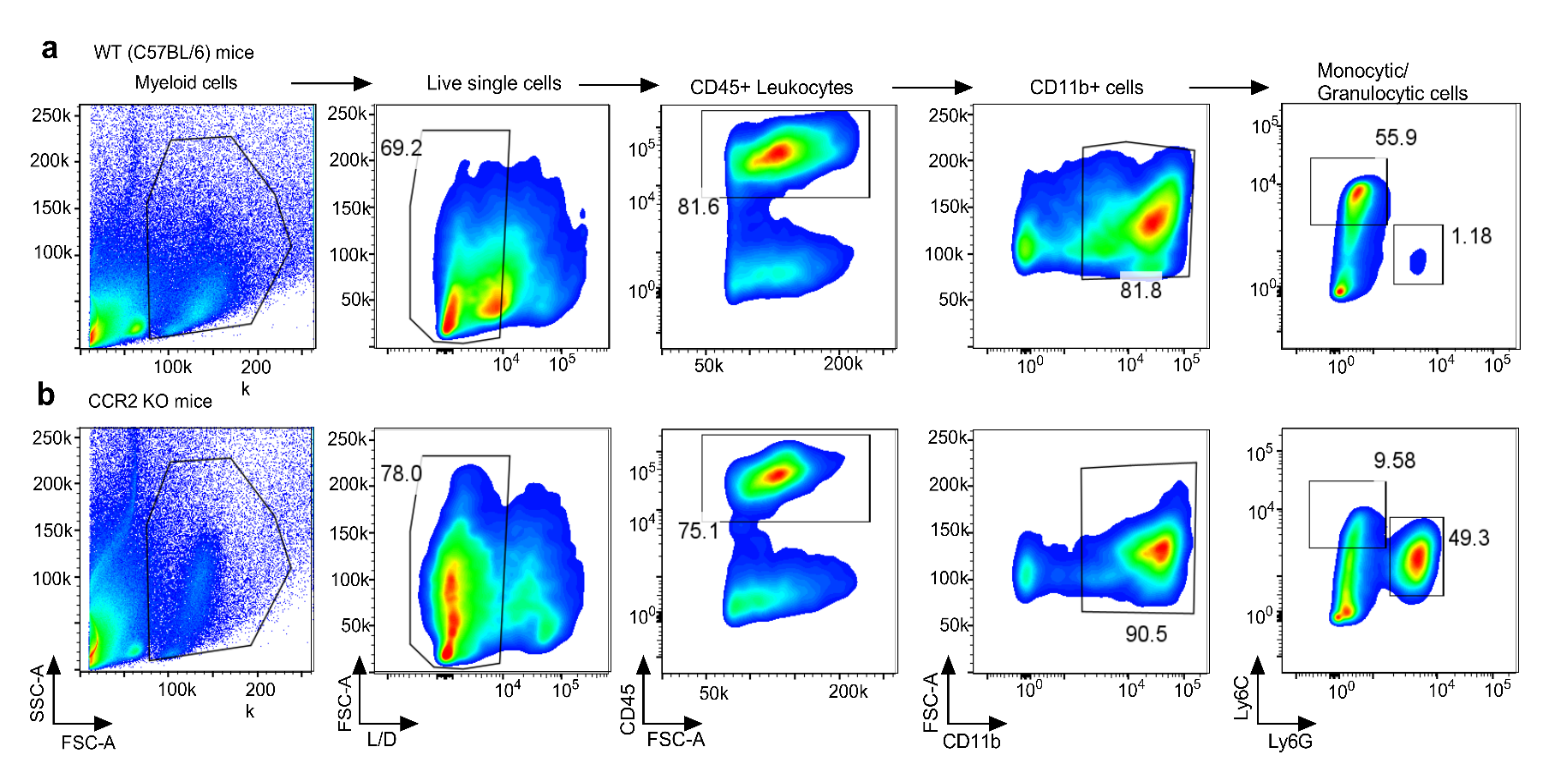


**Gating Strategy for the identification of myeloid cells in the skin of WT and CCR2^-/-^ mice.** Single-cell suspensions were prepared by enzymatic digestion and gentle dissociation of skin from the injection site. Frequencies of CD11b^+^Ly6C^+^ and CD11b^+^Ly6G^+^ cells were determined using the illustrated gating strategy of **a)** WT (C57BL/6J), and **b)** CCR2^-/-^ animals after repeated administrations with ssON for 4 days.

**Figure S3**


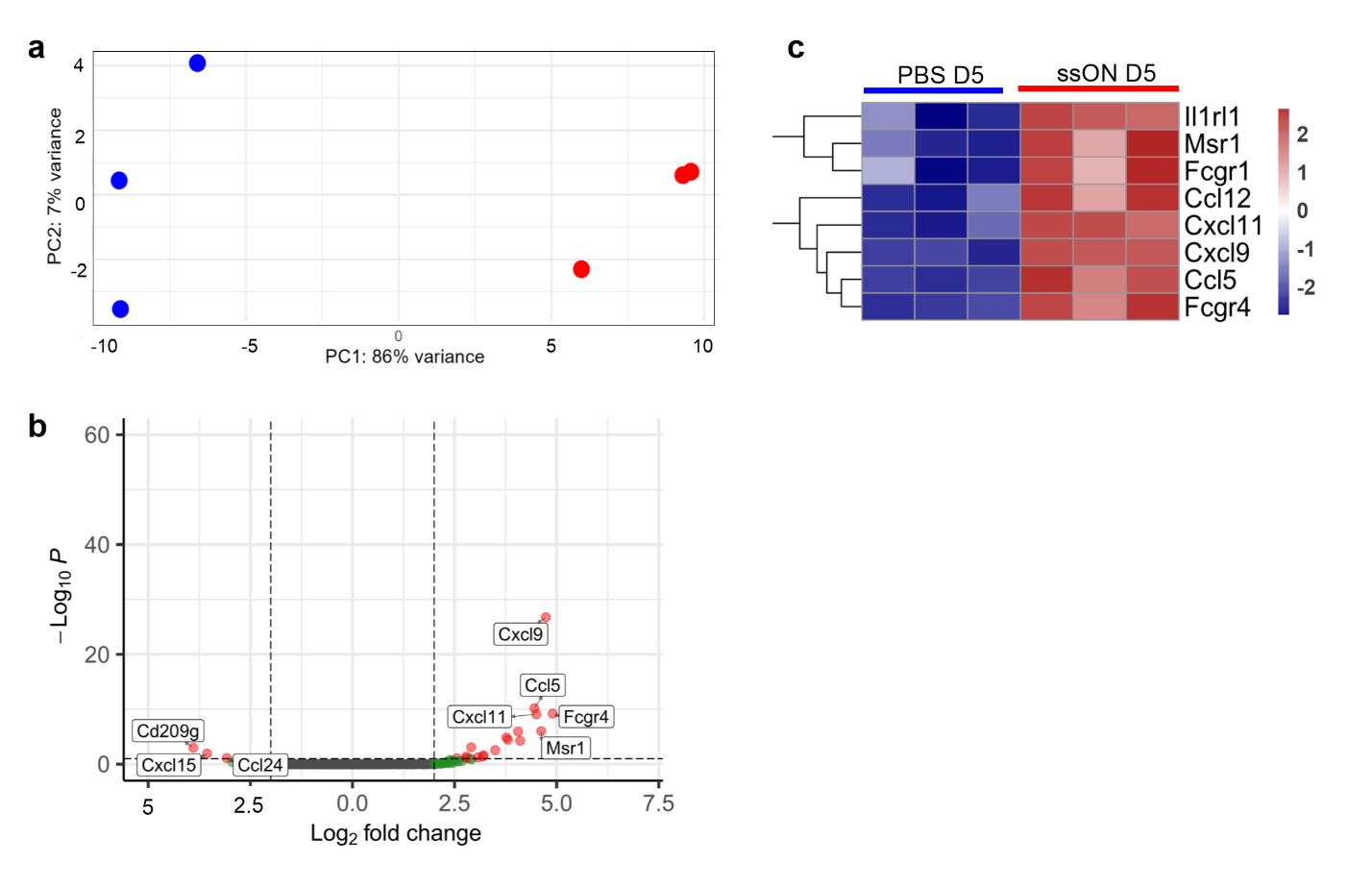


**Transcriptomic analysis of CD45^-^ cells obtained from skin biopsies after repeated ssON administrations. a)** Principal Component analysis depicts the difference between the transcriptome of CD45^-^ cells after treatment with ssON versus PBS. **b)** Volcano plot depicts the differentially expressed genes with Log_2_Fold Change>2 and adjusted *p-*value<0.05. **c**, Heatmap of significantly changed genes across different mice in PBS and ssON group. *n=3* per group.

Figure S4.


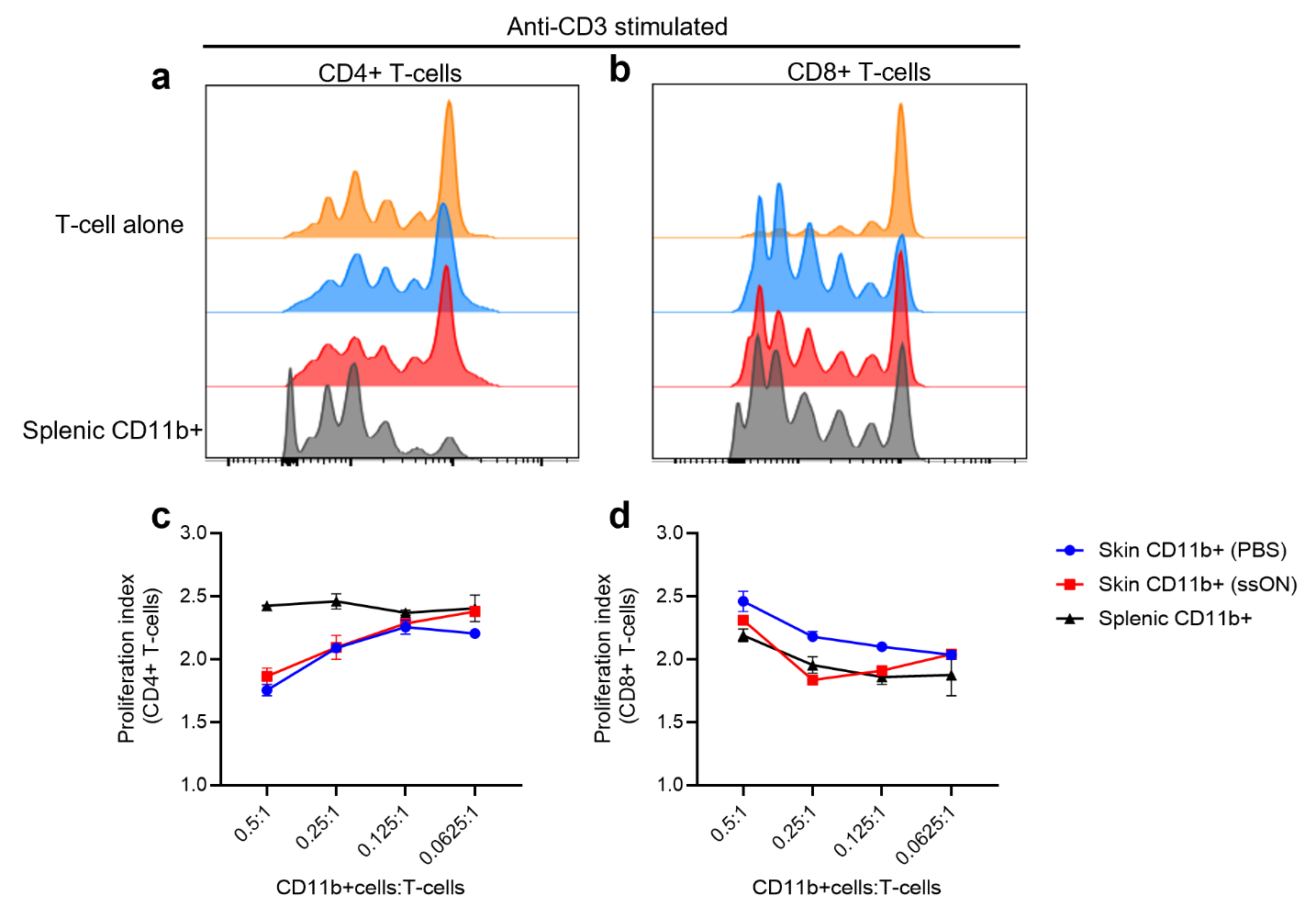


The inhibition of proliferation by skin-derived CD11b^+^ cells can be overcome by the addition of exogenous IL-2. a) Histograms representing proliferating CD4^+^ T-cells after anti-CD3 stimulation in the presence of exogenous IL-2 and with or without indicated APCs. b) Proliferating CD8^+^ T-cells in the presence of IL-2 and with or without indicated APCs. Proliferation indices of c) CD4^+^ T-cells and d) CD8^+^ T-cells at different ratios of CD11b^+^ cells from the different groups in the presence of IL-2. Data is representative of two independent experiments.

**Figure S5.**


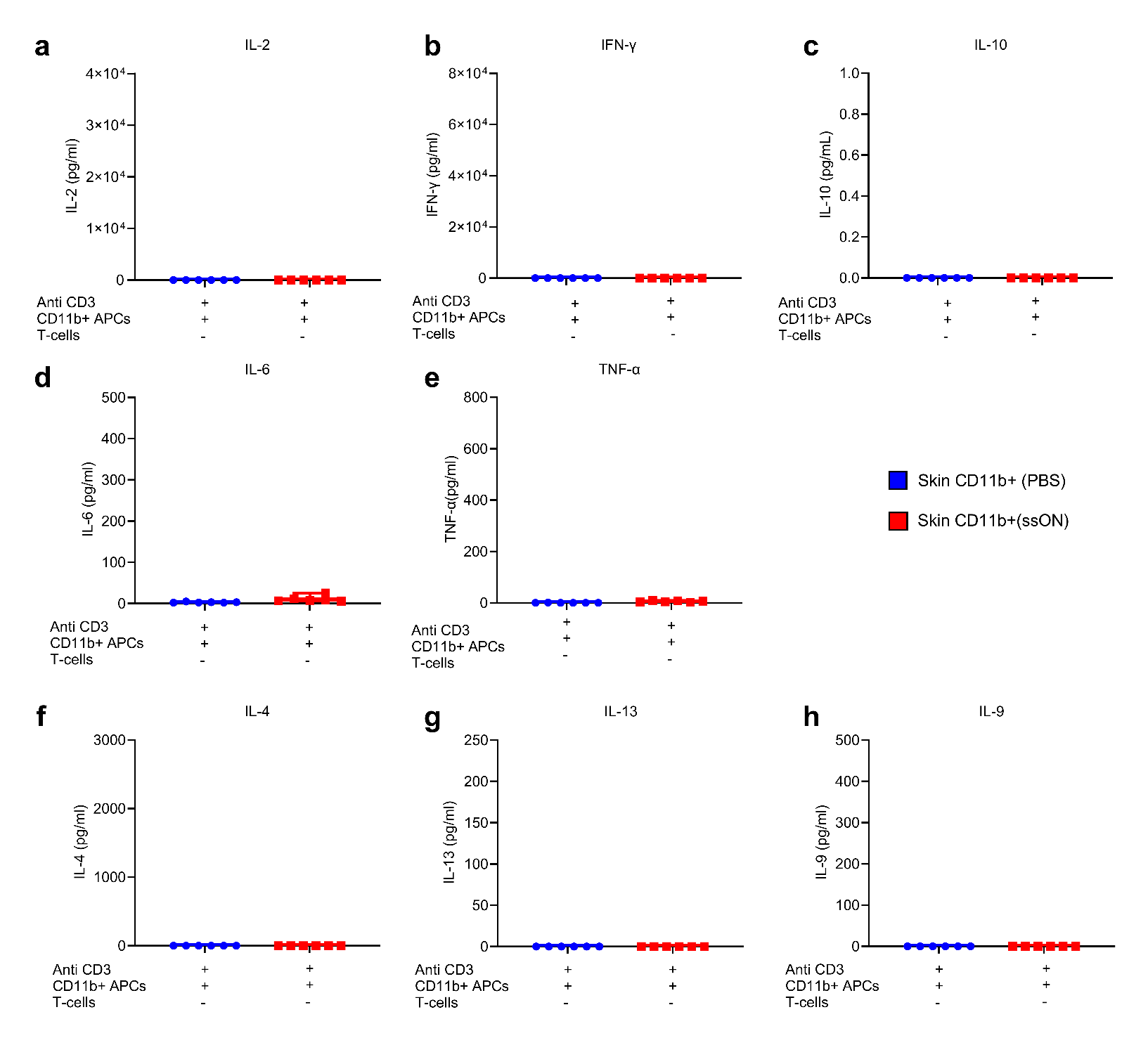


**No detectable cytokine production in co-cultures with skin-derived skin CD11b^+^ cells in the absence of T-cells**

**a)** IL-2 **b)** IFN-γ **c)** IL-10 **d)** IL-6 **e)** TNF-α **f**) IL-4 **g)** IL-13 and **h)** IL-9 production in cell culture supernatants of cutaneous CD11b^+^ cells from either PBS- or ssON-treated mice, in the absence of T-cells. Data are mean±SEM with *n*=6. Statistical analysis was performed using a non-parametric (Mann-Whitney) test.

**Figure S6.**

**Upregulation of *FoxP3* expression on D5 after repeated ssON treatments in the skin**

Upregulation of *FoxP3* mRNA after ssON treatment on D5. A trend toward upregulation of *FoxP3* was observed in the ssON-treated skin samples. Data are mean±SEM from two independent experiments. Statistical analysis was performed using a non-parametric one-way ANOVA test (Kruskal-Wallis test). **p*<0.05.

**Figure S7.**

**
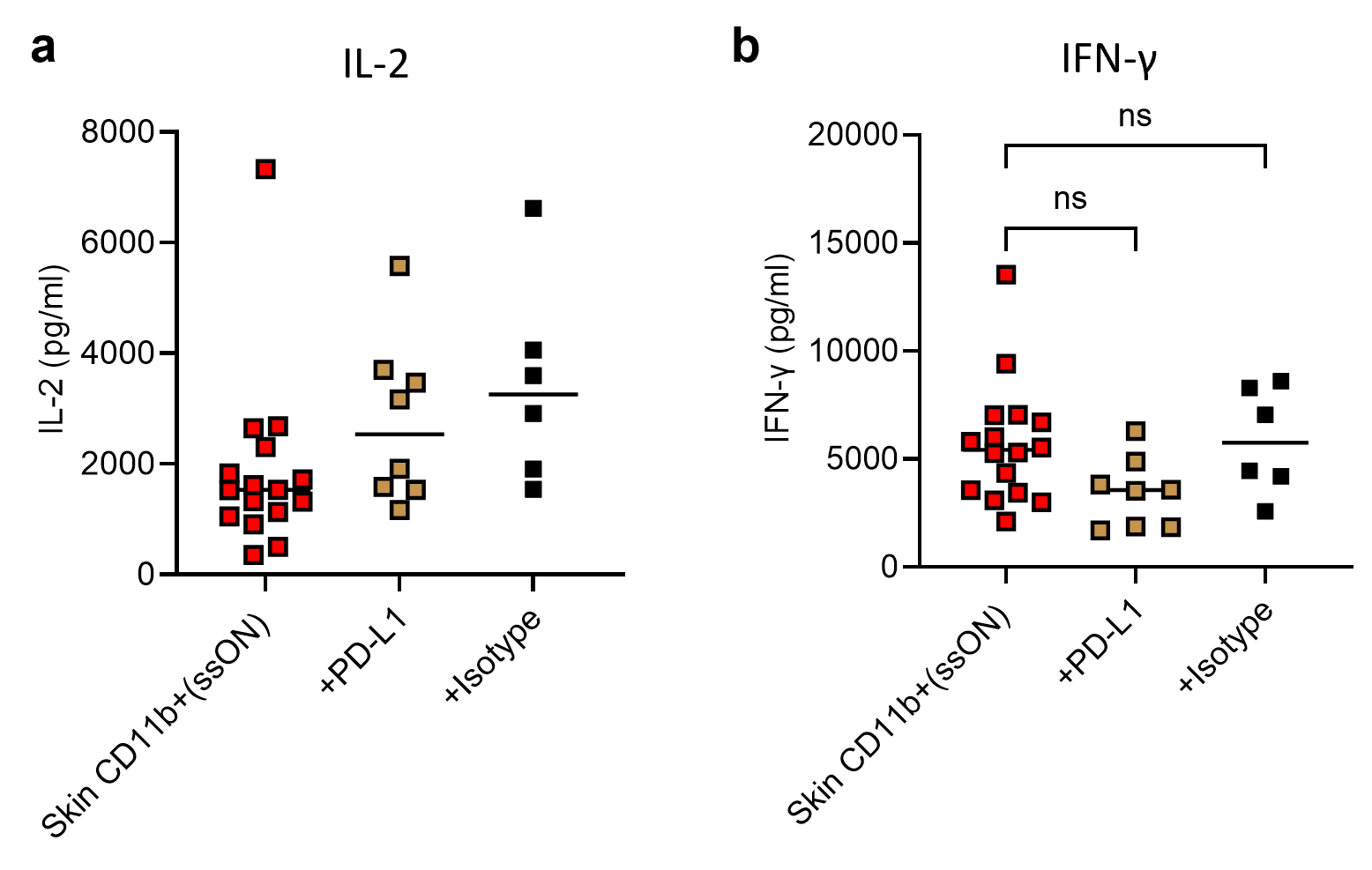
**

**PD-L1 blockade does not affect IL-2 or IFN-γ production in co-cultures of T-cells and CD11b^+^ isolated from ssON-treated skin**

**a**) IL-2 and **b**) IFN-γ production in cell culture supernatants of anti-CD3 activated T-cells co-cultured with cutaneous CD11b^+^ cells from ssON-treated mice, in either the absence or presence of PD-L1 blocking antibody or isotype control. Data are mean±SEM from 3 independent experiments. Statistical analysis was performed using a non-parametric (Mann-Whitney) test.

**Figure S8**


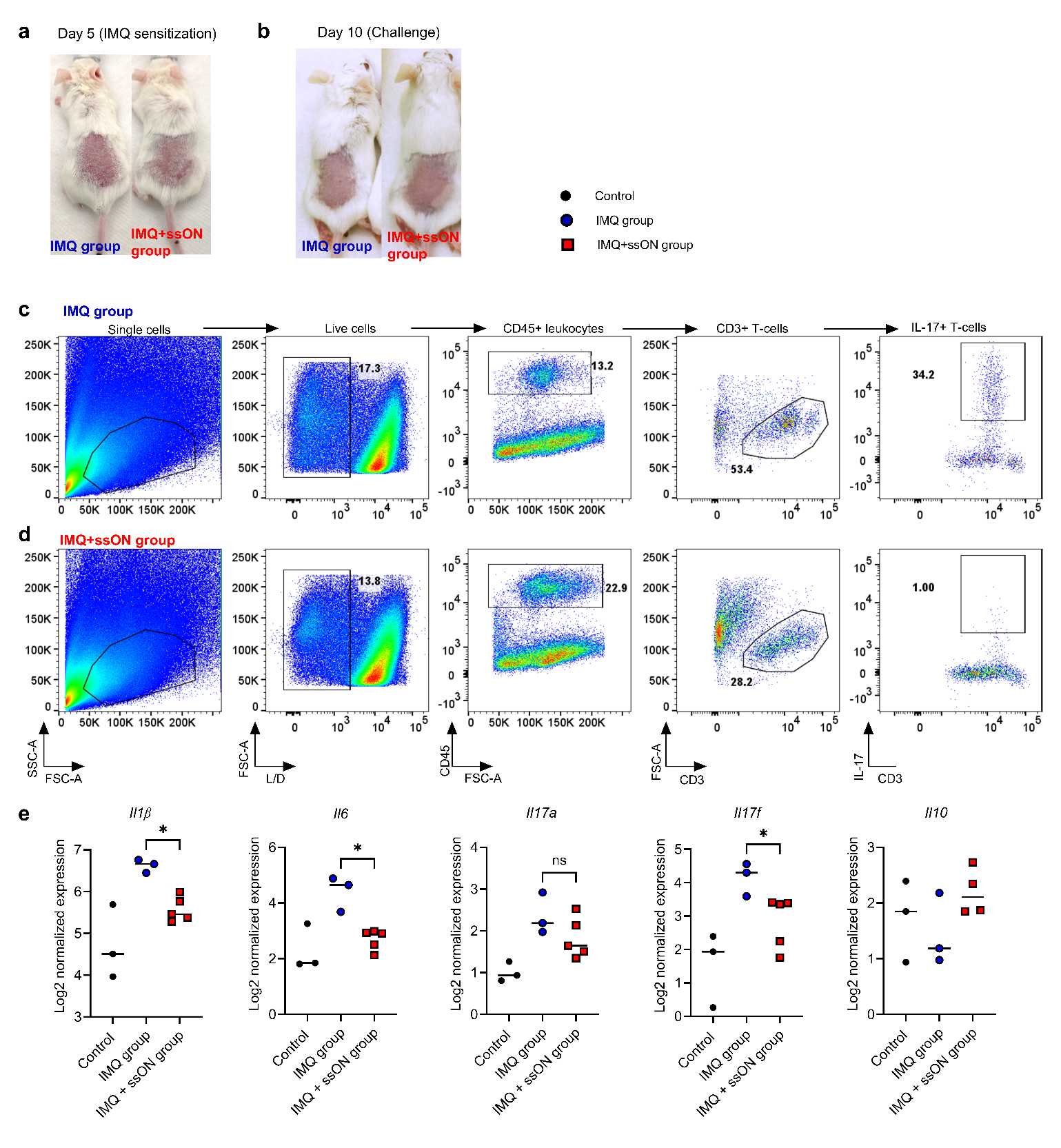


**Effect of ssON treatments in IMQ-mediated psoriasis model**. **a)** Photographs of mice on D5 after treatment with 5% IMQ cream for 5 consecutive days. **b)** Photographs of mice on D10 after challenge with 5% IMQ+ssON administrations. Gating strategy to identify T-cells in the skin that produce IL-17 in **c)** IMQ group **d**) IMQ+ssON group upon ex-vivo stimulation with PMA and Ionomycin. Representative images and plots from two independent experiments. **e)** Log_2_ normalized expression of mRNA levels for inflammatory cytokines *Il1β*, *Il6*, *Il17a*, *IL17f* and *Il10* in the skin biopsies obtained from Control, IMQ and IMQ+ssON groups on D10 by using NanoString analyses. *n*=3-5 in each group. Pairwise comparisons were made using the non-parametric Mann-Whitney test. **p*<0.05.

**Figure S9.**

**
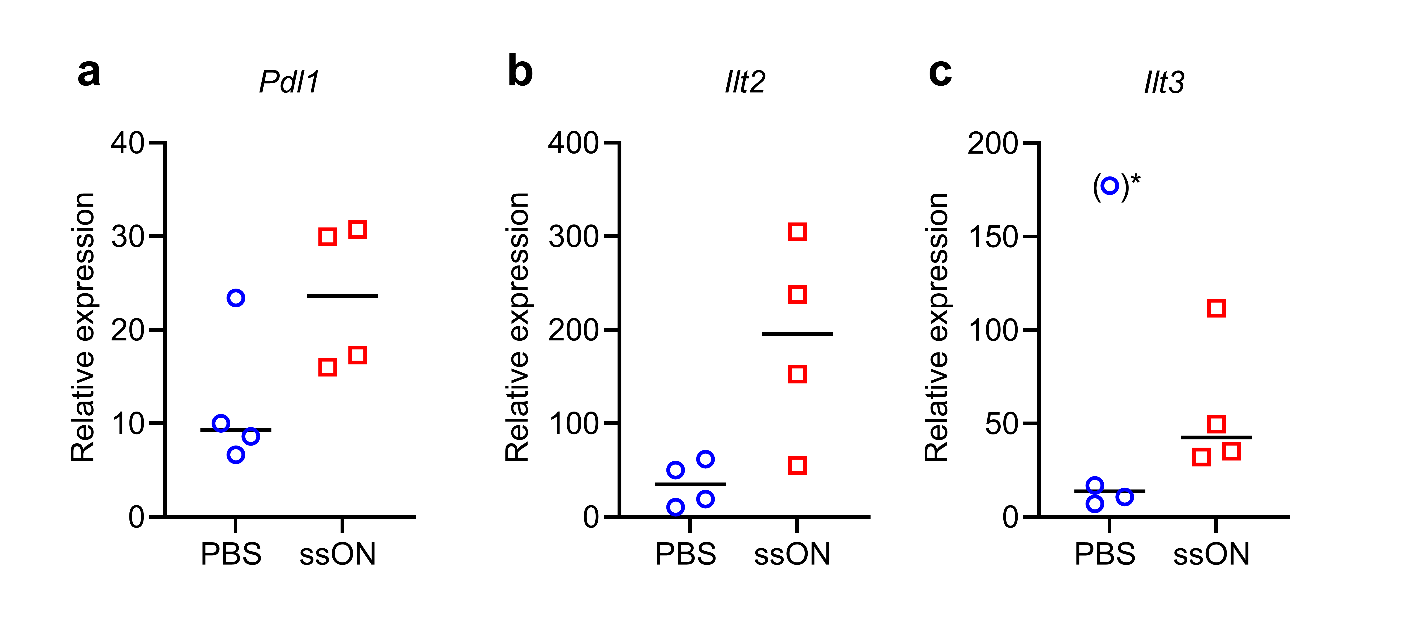
**

**Upregulation of *Pdl1*, *Ilt2* and *Ilt3* expression in macaque skin after ssON treatment.** Relative mRNA expression values of **a)** *Pdl1*, **b*)*** *Ilt2,* and **c)** *Ilt3* from microarray analyses of individual macaque skin biopsies 24 h after injection with PBS or ssON in a published data set by Järver *et al.*^[25]^ Data represented as median with *n*=4. An outlier has been marked with an asterisk in **c**.
